# Chest radiographs in acute respiratory distress syndrome: an Achilles' heel of the Berlin criteria?

**DOI:** 10.3389/fmed.2025.1554752

**Published:** 2025-04-17

**Authors:** Miguel Bardají-Carrillo, Marta Martín-Fernández, Rocío López-Herrero, Juan M. Priede-Vimbela, Irene Arroyo-Hernantes, Rosa Cobo-Zubia, Rosa Prieto-Utrera, Esther Gómez-Sánchez, Jesús Villar, Eduardo Tamayo

**Affiliations:** ^1^BioCritic, Group for Biomedical Research in Critical Care Medicine, Valladolid, Spain; ^2^Anesthesiology and Critical Care, Clinical University Hospital of Valladolid, Valladolid, Spain; ^3^CIBER de Enfermedades Infecciosas (CIBERINFEC), Instituto de Salud Carlos III, Madrid, Spain; ^4^Department of Medicine, Toxicology and Dermatology, University of Valladolid, Valladolid, Spain; ^5^Department of Surgery, University of Valladolid, Valladolid, Spain; ^6^Department of Research and Innovation, Clinical University Hospital of Valladolid (HCUV), SACYL/IECSCYL, Valladolid, Spain; ^7^CIBER de Enfermedades Respiratorias, Instituto de Salud Carlos III, Madrid, Spain; ^8^Research Unit at Hospital Universitario Dr. Negrín, Fundación Canaria Instituto de Investigación Sanitaria de Canarias, Las Palmas de Gran Canaria, Spain; ^9^Li Ka Shing Knowledge Institute at St. Michael's Hospital, Toronto, ON, Canada; ^10^Faculty of Health Sciences, Universidad del Atlántico Medio, Las Palmas de Gran Canaria, Spain

**Keywords:** chest radiographs, postoperative sepsis, acute respiratory distress syndrome, emergency surgery, 60-day mortality

## Abstract

**Background:**

Despite the high mortality and economic burden associated with the acute respiratory distress syndrome (ARDS), the role of chest radiograph (CXR) in ARDS diagnosis and prognosis remains uncertain. The purpose of this study is to elucidate clinical characteristics that distinguish ARDS patients from those without ARDS, especially in patients where CXRs are indicative of ARDS.

**Methods:**

Secondary analysis of a prospective observational study with 454 postoperative septic patients under mechanical ventilation (MV). Patients were stratified in two groups depending on whether they met the Berlin criteria for ARDS. Primary outcome was identification of clinical characteristics differentiating patients with ARDS confirmed by CXR from non-ARDS patients. Secondary outcome was 60-day in-hospital mortality of postoperative sepsis-induced ARDS.

**Results:**

One hundred thirty-nine patients (30.6%) had CXRs compatible with ARDS, although ARDS was confirmed in only 45 patients (9.9%). Emergency surgery (OR 6.6), abdominal source of infection (OR 6.0), pneumonia (OR 8.2), and higher lactate (OR 3.9) were clinical features associated with ARDS development confirmed by CXR. ARDS was an independent risk factor for 60-day mortality (OR 1.8).

**Conclusion:**

Although CXR criteria for ARDS diagnosis could be replaced in future definitions, its importance for ARDS diagnosis should not be underestimated.

## 1 Introduction

With a reported 10% prevalence in patients admitted to intensive care units (ICU), acute respiratory distress syndrome (ARDS) is a medical entity with an associated hospital mortality of about 40% (1). Its mean inpatient costs range from $54,490 to $450,888 in the US (2). Sepsis is one of its main causes (3), and the overall mortality in patients with severe sepsis and ARDS is up to 4-fold higher than septic patients without ARDS (4), being post-operative sepsis-induced ARDS and important but understudied ARDS cause (5).

Recognizing ARDS is not easy. Absence of a specific cause for ARDS and the presence of cardiac failure tend to hinder ARDS diagnosis (1). ARDS diagnosis could be delayed or missed in 66% of patients, with 40% of them without reaching the diagnosis (1), mainly due to interobserver variability in chest radiograph assessment. The Berlin criteria (6) tried to ease this task, although those criteria are still controversial and have low specificity (7). As a result, several authors suggested modifying the ARDS criteria or to outline their limitations (8, 9).

Variability in the chest X-ray (CXR) interpretation is one of the main causes for ARDS misdiagnosis (10), since it has been linked to both underdiagnosis and overdiagnosis (11). Given the burden of sepsis and the challenges associated with ARDS diagnosis, the ability to foresee the risk of developing ARDS is key for achieving early diagnosis and implementing appropriate treatment. To our knowledge, pathological features distinguishing ARDS patients from non-ARDS patients have not been assessed in postoperative septic patients with CXRs compatible with ARDS.

In this report, we aimed to define the clinical characteristics that differentiate patients with radiographic findings of ARDS who have been clinically diagnosed with ARDS, from those patients with radiographic findings compatible with ARDS although they were not diagnosed as having ARDS. We also evaluated the impact of ARDS diagnosis on 60-day mortality.

## 2 Materials and methods

### 2.1 Patient selection

This study is a secondary analysis of a prospective cohort of 454 adult (≥18 years old) patients who underwent major surgery and were admitted to the surgical ICU at the 700-bed Hospital Clínico Universitario de Valladolid in Spain. The study period ranged from December 2006 to February 2017. All participants met the SEPSIS-3 criteria for either sepsis or septic shock (12) and required endotracheal intubation and mechanical ventilation (MV) (13). The study was approved by the hospital Ethics Committee for Clinical Research (approval #PI20-2070). This study followed the Spanish regulations governing biomedical research and adhered to the principles of the Declaration of Helsinki. Prior to enrollment, written informed consent was obtained from all participants, their relatives, or legal representatives.

We excluded patients who met clinical criteria for sepsis or septic shock with a negative microbiological culture, patients on MV for <24h, and patients with ARDS diagnosis prior to surgery. Subsequently, we stratified the cohort of 454 patients into two groups based on the presence or absence of a CXR consistent with ARDS: bilateral opacities on chest X-ray or CT scan that are not fully explained by effusions, lobar/lung collapse, or nodules (6). This categorization was assessed through the evaluation of CXRs by two independent clinicians following the Berlin criteria (6). Discrepancies in eligibility were resolved through discussion and consensus. If a disagreement persisted a third clinician was consulted. Septic patients with CXR compatible with ARDS, according to the Berlin criteria, were stratified into two groups following the Berlin criteria for ARDS diagnosis (6): (i) ARDS and (ii) non-ARDS. When available, CT thoracic scans were reviewed to support ARDS diagnosis.

Throughout the surgical procedures, MV was conducted in accordance with the attending clinician, including adherence to a lung-protective ventilation with a tidal volume (VT) of 6–8 ml/kg predicted body weight and a positive end-expiratory pressure (PEEP) between 6 and 8 cmH_2_O. Recruitment maneuvers were performed after tracheal intubation when deemed necessary and repeated as required by the attending clinician. We followed existing guidelines for general critical care practices (12), which include: (i) early identification of causative microorganism, optimization of intravenous antibiotic selection and timely administration based on the antibiogram; (ii) fluid resuscitation and vasopressor use were individualized for maintaining a systolic blood pressure ≥90 mmHg or a mean arterial pressure ≥65 mmHg; and (iii) maintenance of hemoglobin between 7 and 10 g/dL (14). Selection of medications for sedation and analgesia and the approach to hemodynamic treatment were at the discretion of attending clinicians. Weaning from the ventilator began when deemed clinically appropriate by the attending physician. Gastric protection was routinely performed with omeprazole (40 mg/iv) during the first 24 h of ICU stay.

### 2.2 Data collection and follow-up

During the study period, patients admitted to ICU underwent daily screening to evaluate the development of sepsis or septic shock. A specialized standardized form was used to collect demographic and clinical information, including hematological, biochemical, radiological, microbiological, and biomarker data, all recorded within the initial 24 h following the diagnosis of sepsis or septic shock. Disease severity was assessed using the Sequential Organ Failure Assessment (SOFA) scale (15) and the Acute Physiology and Chronic Health Evaluation II (APACHE II) (16) score. Sepsis was defined as a life-threatening organ dysfunction (indicated by an increase in SOFA score ≥2 points) resulting from an abnormal host response to infection (12). Septic shock was recognized by the need of vasopressors to maintain a mean arterial pressure ≥65 mmHg and serum lactate >2 mmol/L (>18 mg/dL) in the absence of hypovolemia. After verifying that no patient was infected prior to the surgical procedure, we followed the criteria of Centers for Disease Control and Prevention (CDC) (17) for the diagnosis of nosocomial infections during ICU stay. ARDS was diagnosed according to Berlin criteria (6), which include: (i) hypoxemia occurring within 1 week of a well-known clinical insult or a further exacerbation of respiratory symptoms, (ii) bilateral opacities on CXRs that are not attributable to pleural effusions, lobar or pulmonary collapse, and (iii) acute respiratory failure not fully accounted either by cardiac insufficiency or fluid overload. For excluding patients with heart failure as a cause of pulmonary edema, echocardiographic images (when available), clinical history, or pulmonary arterial monitoring data were assessed. Patients with dobutamine >5 μg/kg/min or levosimendan infusion were excluded as ARDS patients, being assumed as heart failure. PaO_2_/FiO_2_ ratios were recorded at the time of ARDS diagnosis, as mandated by the Berlin definition.

### 2.3 Clinical endpoints and statistical analysis

The primary endpoint was to define clinical characteristics or features that differentiate patients with CXR findings consistent with ARDS who truly developed ARDS. The secondary endpoint was to assess the influence of ARDS on 60-day in-hospital mortality.

Differences between groups were assessed using the Chi-square test for categorical variables and the Mann Whitney U test for continuous variables. Categorical variables were expressed in percentages, while continuous variables were expressed as median [interquartile range (IQR)]. Potential association between clinical variables and ARDS were evaluated using a Wald backward stepwise multivariate logistic regression analysis. Potential confounding factors for logistic regression were identified from variables described in Table 1 and Supplementary Tables S1–S3. In the ARDS analysis, variables yielding a *p* < 0.1 in the univariate regression analysis were included in the multivariate analysis as adjusting variables [cancer, emergency surgery, Napierian logarithm of lactate and procalcitonin, abdominal infection, pneumonia, and APACHE score >15]. In the 60-day mortality analysis, variables yielding a *p* < 0.1 in the univariate regression analysis were included in the multivariate analysis as adjusting variables [age, sex, SOFA score >8, ARDS]. We analyzed the probability of death to day 60 after sepsis diagnosis using Kaplan–Meier curves and tested with the log-rank test (Mantel–Haenszel). We considered 2-sided *p* < 0.05 to indicate statistical significance. Statistical power was 99.9% with 95% confidence. All data were analyzed using the IBM SPSS 26.0 software (SPSS, Chicago, IL).

**Table 1:** Preoperative and postoperative features at baseline based on the presence of ARDS.

	**Non-ARDS (*n* = 94)**	**ARDS (*n* = 45)**	***p*-value**
**Characteristics**			
Age [years, median (IQR)]	76 [13]	76 [17]	0.793
Male [%, (n)]	62.1% (59)	57.8% (26)	0.735
**Comorbidities, [% (n)]**			
Chronic cardiovascular disease	40% (38)	22.2% (10)	**0.046**
Chronic respiratory disease	20% (19)	17.8% (8)	0.565
Chronic renal failure	10.6% (10)	6.7% (3)	0.661
Diabetes mellitus	22.1% (21)	24.4% (11)	0.706
Cancer	23.2% (22)	46.7% (21)	**0.004**
Obesity	22.1% (21)	11.1% (5)	0.131
Smoker	17.5% (17)	20% (9)	0.719
**Surgery type, [% (n)]**			
Abdominal	60.6% (57)	88.9% (40)	**0.001**
Cardio-thoracic	16% (15)	0% (0)	**0.005**
Vascular	8.5% (8)	6.7% (3)	0.706
Urological/renal	8.5% (8)	2.2% (1)	0.159
Other	5.3% (5)	2.2% (1)	0.401
Emergency surgery	20.2% (19)	73.3% (33)	**<0.001**
**Source of infection, [% (n)]**			
Pneumonia	13.8% (13)	42.2% (19)	**<0.001**
Abdomen	47.9% (45)	75.6% (34)	**0.002**
Urinary tract	5.3% (5)	2.2% (1)	0.401
Surgical site	1.1% (1)	2.2% (1)	0.592
Bacteremia	2.1% (2)	2.2% (1)	0.971
Other	10.9% (10)	8.9% (4)	0.719
**Microbiology [% (n)]**			
Gram +	27.4% (26)	11.1% (5)	**0.035**
Gram –	36.8% (35)	40% (18)	0.839
Fungi	22.1% (21)	15.6% (7)	0.397
**Severity scores [median (IQR)]**			
SOFA score [median (IQR)]	9 [4]	9 [2]	0.455
SOFA score > 8 [% (n)]	55.8% (53)	65.9% (29)	0.259
APACHE II score [median (IQR)]	16 [8]	17 [5]	0.158
APACHE II score > 15 [% (n)]	52.6% (50)	70.5% (31)	**0.047**
**Time course and outcomes**			
Length of MV (days) [median, (IQR)]	5.50 [16]	6 [14]	**0.035**
Length of hospital stay (days) [median, (IQR)]	25 [32]	30 [27]	0.784
Length of ICU stay (days) [median, (IQR)]	9.5 [17]	14 [18]	**0.041**
Septic shock [% (n)]	84.2% (80)	93.3% (41)	0.143
Mortality at 60 days [%, (n)]	34.7% (33)	55.6% (25)	**0.014**
In-hospital mortality [%, (n)]	32.6% (31)	51.1% (23)	**0.027**
Bronchial aspiration [%, (n)]	1.1% (1)	8.9% (4)	**0.020**

Continuous variables are represented as median and interquartile range (IQR); categorical variables are represented as percentages (%) and number (n). SOFA, sequential organ failure assessment; APACHE, Acute Physiology and Chronic Health Evaluation; MV, mechanical ventilation; ICU, intensive care unit. Bold values show statistically significant *p*-values.

## 3 Results

**Figure 1:**
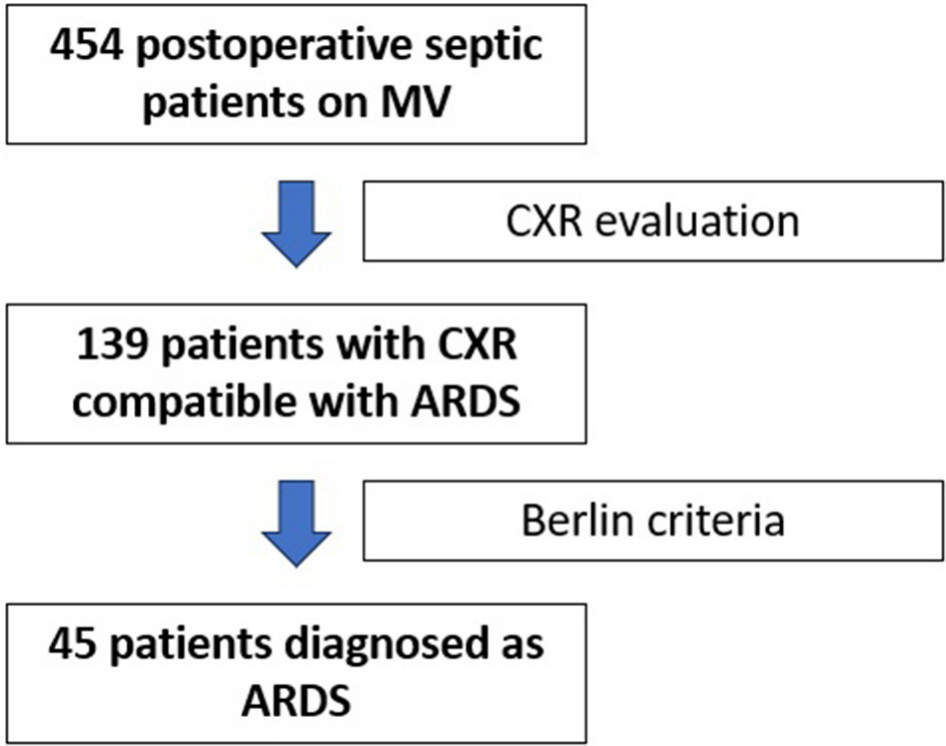
Study flowchart. MV, mechanical ventilation; CXR, chest radiograph; ARDS, acute respiratory distress syndrome.

A total of 139 patients (30.6%) out of 454 patients of our cohort, had a CXR compatible with ARDS. However, only 45 patients of them (32.3%) were diagnosed as having ARDS by the Berlin criteria (6), while 94 patients (67.6%) were not diagnosed as ARDS. Figure 1 shows the study flowchart. Among the 45 ARDS patients, eight had a CT thoracic scan, and all showed images consistent with ARDS. Comprehensive baseline characteristics at the onset of the study are reported in Table 1 and Supplementary Tables S1–S3.

Patients with ARDS had a higher prevalence of cancer as a comorbidity [46.7 vs. 23.4%, *p* = 0.004], whereas chronic cardiovascular disease was less prevalent [22.2 vs. 40%, *p* = 0.046] (Table 1). We observed a higher prevalence of abdominal surgery [88.9 vs. 60.6%, *p* < 0.001] and emergent surgical interventions [73.3 vs. 20.2%, *p* < 0.001] among ARDS patients (Table 1). Bronchial aspiration was more frequent in ARDS than in non-ARDS [6.9 vs. 1.2%, *p* = 0.020] (Table 1).

**Figure 2:**
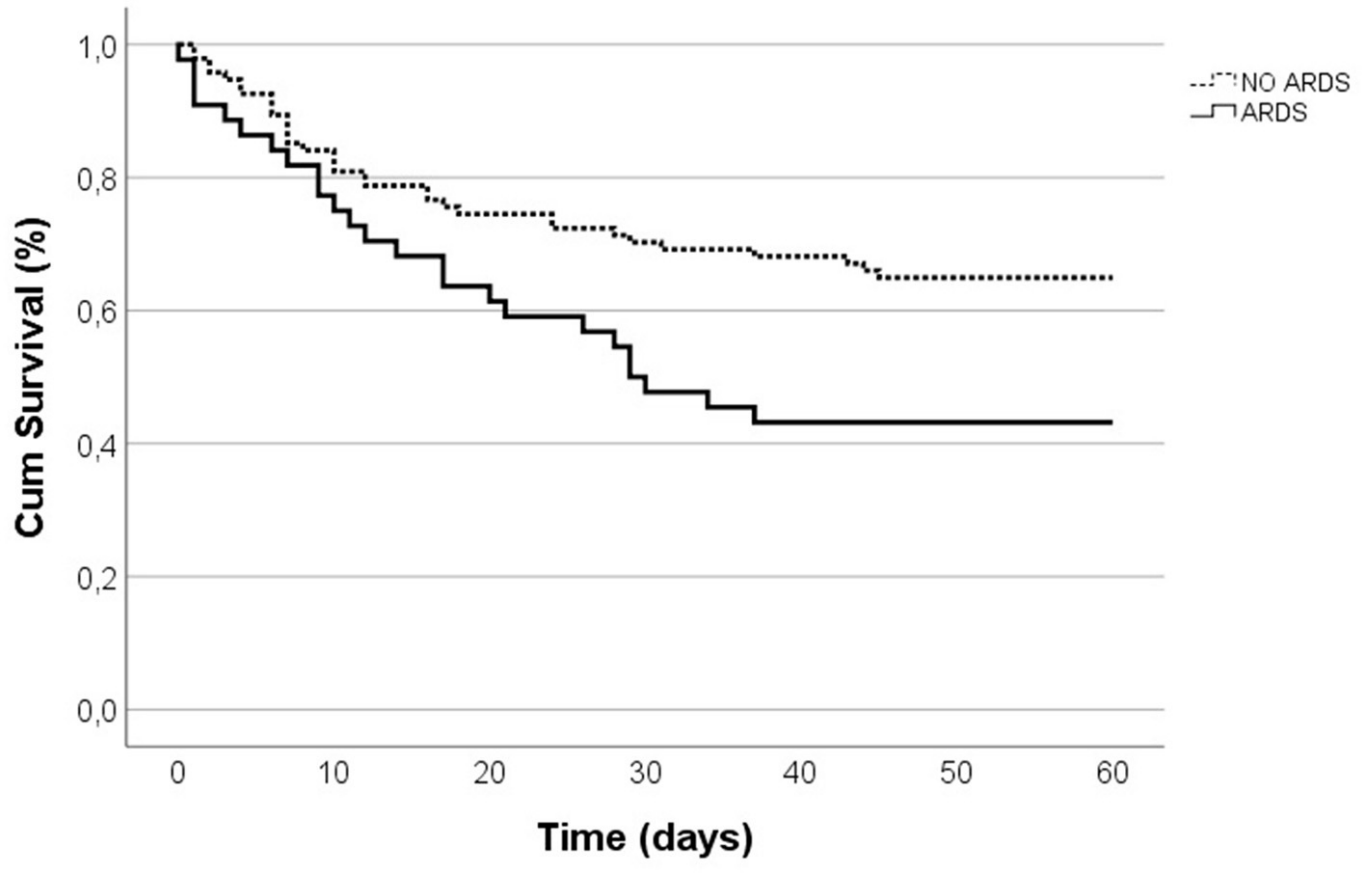
Kaplan-Meier survival curves for 60-day in-hospital mortality.

Patients with ARDS had a high prevalence of abdominal infections [75.6 vs. 47.9%, *p* = 0.002] and pneumonia [42.2 vs. 13.8%, *p* < 0.001] (Table 1) as the underlying cause. Patients with ARDS had a greater APACHE II scores exceeding 15 points [70.5 vs. 52.6%, *p* = 0.047], prolonged ICU stay [14 (18) vs. 9.5 (17), *p* = 0.041], and extended periods of MV [6 (14) vs. 5.5 (16), *p* = 0.035] (Table 1). This cohort had reduced compliance [22.45 (20.95) vs. 30.5 (10.25), *p* = 0.006] and elevated driving pressure [20 (9) vs. 17.5 (7), *p* = 0.008] (Supplementary Table S1). Additionally, lower arterial pH [7.31 (0.15) vs. 7.36 (0.13), *p* = 0.046], elevated lactate levels [4.6 (3.33) vs. 2.3 (1.8), *p* < 0.001], and heightened procalcitonin levels [21.5 (58.37) vs. 4.1 14.2), *p* = 0.011] were more frequent in ARDS (Supplementary Table S1). The 60-day mortality was higher in ARDS patients [55.6 vs. 34.7%, *p* = 0.014] (Table 1). On average, patients with ARDS died earlier when assessing 60-day mortality (log-rank *p* = 0.016; Figure 2). Emergency surgery (OR 6.60, 95%CI 2.29–18.90, *p* < 0.001), abdominal source of infection (OR 5.97, 95%CI 1.77–20.19, *p* = 0.004), pneumonia (OR 8.15, 95%CI 2.33–28.47, *p* = 0.001) and higher lactate (OR 3.94, 95%CI 1.30–11.87, *p* = 0.015) were independently associated with ARDS development (Table 2).

**Table 2:** Multivariate analysis for evaluating the risk of ARDS development.

	**OR**	**[CI 95%]**	***p*-value**
Emergency surgery	6.57	2.29–18.90	**<0.001**
Abdominal source of infection	5.97	1.77–20.19	**0.004**
Pneumonia	8.15	2.33–28.47	**0.001**
Lactate Ln	3.93	1.30–11.87	**0.015**

Bold values show statistically significant *p*-values.

A total of 58 ARDS patients (41.7%) died by day 60, while 81 (58.3%) survived. Non-survivors were characterized by advanced age compared to survivors [78 (10) vs. 74 (16), *p* = 0.007]. Emergency surgery was more frequent in non-survivors [55.5 vs. 24.7%, *p* < 0.001] (Supplementary Table S2). Pneumonia was more prevalent in non-survivors [36.2 vs. 13.6%, *p* = 0.002], accompanied by higher SOFA [10 (4) vs. 8 (4), *p* = 0.001] and APACHE II [18 (6) vs. 15 (6), *p* = 0.001] scores (Supplementary Table S2). Non-survivors also experienced longer hospital stay [30 (27) vs. 25 (32), *p* = 0.016] and prolonged MV duration [5 (14) vs. 1 (10), *p* = 0.007] (Supplementary Table S2). The incidence of septic shock [96.6 vs. 80.2%, *p* = 0.005] and ARDS [43.1 vs. 23.5%, *p* = 0.014] was higher in non-survivors (Supplementary Table S2). Furthermore, non-survivors had lower arterial pH [7.31 (0.15) vs. 7.36 (0.13), *p* < 0.001], higher lactate levels [3 (2.9) vs. 2.2 (1.9), *p* = 0.010], and elevated procalcitonin levels [9.9 (40.3) vs. 3.3 (13.7), *p* = 0.005] (Supplementary Table S3).

In the multivariate analysis, ARDS remained independently associated with 60-day mortality (OR 1.81, 95%CI 1.06–3.07, *p* = 0.029), along with older age (OR 1.04, 95%CI 1.01–1.08) and a SOFA score >8 (OR 2.00, 95%CI 1.12–3.59, *p* = 0.019; Table 3).

**Table 3:** Multivariate analysis for evaluating the risk of 60-day in-hospital mortality.

	**OR**	**[CI 95%]**	***p*-value**
Age	1.04	1.01–1.08	**0.006**
ARDS	1.81	1.06–3.07	**0.029**
SOFA >8	2.00	1.12–3.59	**0.019**

Bold values show statistically significant *p*-values.

## 4 Discussion

In this cohort of 454 postoperative patients who developed sepsis or septic shock, our most relevant findings were: (i) 139 (30.6%) had a CXR compatible with ARDS although only 45 of them (9.9%) were diagnosed as ARDS; (ii) emergency surgery, abdominal source of infection, pneumonia, and higher lactate levels were clinical features differentiating patients with ARDS; (iii) ARDS was associated with prolonged ICU stay, longer duration of MV and increased in-hospital mortality by 1.8-fold.

While sepsis represents a third of ARDS etiology (18), the prevalence of ARDS in septic patients varies between 7 and 14% (19), and they experience a worse prognosis (18). Despite 30.6% of patients having CXR consistent with ARDS, the prevalence of ARDS in our 454 patient's cohort (9.9%) aligns with existing literature. Differential diagnosis between ARDS and other causes of pulmonary edema (including heart failure, fluid overload, severe atelectasis, or severe pleural effusion) can be challenging, as similar radiologic features and hypoxemia may be seen in the setting of those entities (20). Although there is not many published reports comparing ARDS and cardiogenic pulmonary edema, Schmickl et al. (21) reported that higher severity of illness, pneumonia or chemotherapy (in the context of cancer) are more frequent in ARDS patients compared to cardiogenic pulmonary edema patients. Gastric aspiration was also linked to ARDS development (21), although it did not reach statistical significance in our cohort. History of heart failure or coronary artery disease are significantly less frequent in ARDS patients (22), as they can cause cardiogenic pulmonary edema (23). Early diagnosis is essential to start adequate management and treatment as soon as possible. Also, it is of paramount importance to know potential ARDS risk factors. From our findings, we found that emergency surgery, abdominal source of infection, pneumonia, and higher lactate are clinical characteristics that should be considered ARDS risk factors in critically ill patients. Although CXR sensitivity for ARDS diagnosis cannot be altered, we have identified several clinical features that could assist clinicians in the diagnosis of ARDS when there is uncertainty about CXR.

Although the Berlin criteria have lost supporters (8, 9), CXR remains to be one of the main criteria for ARDS diagnosis (6, 24). This main criterion has been lately much criticized due to a high interobserver variability. Recognition of CXR consistent with ARDS can range from 51% in mild ARDS to 79% in severe cases (25). After evaluating the CXR in each patient of our cohort, three independent observers identified 139 (30.6%) CXRs indicative of ARDS, although only 45 patients (9.95%) did truly develop ARDS. In recent years, ultrasonography and CT scans have shown higher sensitivity and specificity for ARDS (26), although with inherent limitations (25). Therefore, although the CXR criterion could be an Achilles' heel of the Berlin criteria, this criterion is still necessary to diagnose ARDS. Machine learning is also gaining adepts in ARDS diagnosis (26, 27). In our cohort of 45 ARDS patients, eight of them had a CT thoracic scan, and all showed images consistent with ARDS. Although the number of patients with a CT thoracic scan in our cohort was limited, in the coming years, they are poised to potentially replace CXR, especially considering their potential to exhibit lower interobserver variability.

Mortality among ARDS patients is significantly higher compared to those with cardiogenic pulmonary edema: age and severity of illness were independent predictors for mortality (21, 22), and the mortality risk in the subset of septic ARDS surpasses that observed in ARDS cases originating from alternative etiologies (18). The severity of CXR findings is also correlated with increased mortality (28), showing the importance of CXR assessment in patients suspected of having ARDS. However, the LUNG SAFE (29) and the PANDORA (30) studies showed that unilateral or bilateral infiltrates in ARDS patients have similar outcomes. ICU length of stay is longer in ARDS patients (1, 21), with the economic burden that it entails. Early recognition of risk factors would allow a reduction in mortality and hospital stay, with an early initiation of targeted treatment.

We acknowledge that our study has potential limitations. First, given the retrospective nature of our study design, there is potential misclassification of some patients based on the available CXR and blood gas measurements. However, we based the definition of ARDS on the Berlin criteria (6) and verified the accuracy of our assessment by a separate case review by an independent physician investigator. Second, a larger population would be needed to confirm that other variables could be associated with ARDS development, such as cancer or differences in compliance and driving pressure, since in our cohort only 45 patients developed ARDS. Third, although we assessed several CXRs per patient to evaluate whether their CXR was compatible with ARDS, we did not assess its development and we did not evaluate its evolution in 12–24 h period. Fourth, we only focus on postoperative sepsis induced-ARDS. Fifth, further studies should be conducted to assess whether other diagnostic tools are more accurate and accessible than CXR for ARDS diagnosis.

In conclusion, although CXR criteria for ARDS diagnosis could be replaced by alternative diagnostic tools with lower interobserver variability in an updated definition, its importance for ARDS diagnosis should not be underestimated. Postoperative septic patients with CXR consistent with ARDS who associate clinical characteristics linked to ARDS development, such as pneumonia, abdominal infection, emergency surgery, or higher lactate levels, should be carefully monitored. ARDS was independently associated with 60-day in-hospital mortality.

## Data Availability

The raw data supporting the conclusions of this article will be made available by the authors, without undue reservation.
