# Supplementary material for: Chest radiographs in acute respiratory distress syndrome: an Achilles' heel of the Berlin criteria?
Source: Front Med (Lausanne). 2025 Apr 17;12:1554752. doi: 10.3389/fmed.2025.1554752 (PMC12043692; doi:10.3389/fmed.2025.1554752)
Supplement: Supplementary file 1 [file Table_1.docx]

Supplementary Material

# Supplementary Figures and Tables

**Table S1. Measurements at diagnosis based on ARDS.**

|  | **Non-ARDS** (n=94) | **ARDS**  (n=45) | **p-value** |
| --- | --- | --- | --- |
| **­Measurements at diagnosis,** [median [IQR]] |  |  |  |
| Mean arterial pressure (mmHg) | 66 [12] | 63 [11] | 0.681 |
| Hear rate (bpm) | 110 [30] | 120 [38] | **0.010** |
| PaCO_2_ (mmHg) | 41.5 [11.2] | 40 [13.8] | 0.411 |
| SaO_2_ (%) | 95.2 [5.9] | 94.4 [6.8] | 0.539 |
| PaO_2_ (mmHg) | 86.7 [43.3] | 82.9 [42.7] | 0.178 |
| pH | 7.36 [0.13] | 7.31 [0.15] | **0.046** |
| HCO_3_ (mmol/L) | 22.2 [5.8] | 21.5 [8.8] | 0.749 |
| Creatinine (mg/mL) | 1.93 [3.13] | 1.90 [1.88] | 0.865 |
| LDH (UI) | 320 [192] | 214 [177] | **0.019** |
| Troponins (UI) | 43.05 [826.56] | 34.99 [595] | 0.184 |
| Lactate (mmol/L) | 2.3 [1.8] | 4.6 [3.33] | **<0.001** |
| Procalcitonin (ng/ml) | 4.1 [14.2] | 21.5 [58.37] | **0.011** |
| C-Reactive Protein (mg/L) | 243 [188] | 247.3 [241.93] | 0.244 |
| White Blood cells (cells/mm^3^) | 14270 [10834] | 10000 [13052] | 0.207 |
| Compliance (mL/cmH_2_O) | 30.5 [20.95] | 22.45 [10.25] | **0.006** |
| Driving pressure (cmH_2_O) | 17.50 [7] | 20 [9] | **0.008** |

*Continuous variables are represented as median and interquartile range (IQR). LDH: Lactate Dehydrogenase*

**Table S2. Preoperative and postoperative features based on 60-day in-hospital mortality.**

|  | **60-day Survivors (n=81)** | **60d nonsurvivors (n=58)** | **p-value** |
| --- | --- | --- | --- |
| **Characteristics** |  |  |  |
| Age [years, median [IQR]] | 74 [16] | 78 [10] | **0.007** |
| Male [%, (n)] | 59.3% (48) | 63.8% (37) | 0.589 |
| Comorbidities, [% (n)] |  |  |  |
| Chronic cardiovascular disease | 35.8% (29) | 32.8% (19) | 0.710 |
| Chronic respiratory disease | 19.8% (16) | 17.2% (10) | 0.708 |
| Chronic renal failure | 9.8% (8) | 10.5% (6) | 0.660 |
| Diabetes mellitus | 21% (17) | 25.9% (15) | 0.501 |
| Cancer | 32.1% (26) | 29.3% (17) | 0.726 |
| Obesity | 19.8% (16) | 17.2% (10) | 0.708 |
| Smoker | 23.5% (19) | 12.1% (7) | 0.090 |
| **Surgery type, [% (n)]** |  |  |  |
| Abdominal | 65.4% (53) | 75.9% (44) | 0.187 |
| Cardio-thoracic | 14.8% (12) | 5.2% (3) | 0.071 |
| Vascular | 7.4% (6) | 8.6% (5) | 0.794 |
| Urological/Renal | 7.4% (6) | 5.2% (2) | 0.597 |
| Other | 2.5% (2) | 6.9% (4) | 0.205 |
| Emergency surgery | 24.7% (20) | 55.2% (32) | **<0.001** |
| **Source of infection, [% (n)]** |  |  |  |
| Pneumonia | 13.6% (11) | 36.2% (21) | **0.002** |
| Abdomen | 54.3% (44) | 60.3% (35) | 0.480 |
| Urinary tract | 4.9% (4) | 3.4% (2) | 0.670 |
| Bacteremia | 1.2% (1) | 3.4% (2) | 0.376 |
| Surgical site | 1.2% (1) | 1.7% (1) | 0.811 |
| Other | 11.1% (9) | 8.9% (5) | 0.678 |
| **Microbiology [% (n)]** |  |  |  |
| Gram + | 21% (17) | 24.1% (14) | 0.660 |
| Gram - | 35.8% (29) | 39.7% (23) | 0.643 |
| Fungi | 20.4% (20) | 13.8% (8) | 0.114 |
| **Severity scores** |  |  |  |
| SOFA score [median [IQR]] | 8 [4] | 10 [4] | **0.001** |
| SOFA score > 8 [% (n)] | 49-4% (40) | 72.4% (42) | **0.006** |
| APACHE II score [median [IQR]] | 15 [6] | 18 [6] | **0.001** |
| APACHE II score > 15 [% (n)] | 49.4% (40) | 70.7% (41) | **0.012** |
| **Time course and outcomes** |  |  |  |
| Length of MV (days) [median, [IQR]] | 1 [10] | 5 [14] | **0.007** |
| Length of hospital stay (days) [median, [IQR]] | 25 [32] | 30 [27] | **0.016** |
| Length of ICU stay (days) [median, [IQR]] | 10 [13] | 10.5 [18] | 0.424 |
| Septic shock [%, (n)] | 80.2% (65) | 96.6% (56) | **0.005** |
| Bronquial aspiration [%, (n)] | 1.2% (1) | 6.9% (4) | 0.077 |
| ARDS [%, (n)] | 23.5% (19) | 43.1% (25) | **0.014** |

*Continuous variables are represented as median and interquartile range (IQR); categorical variables are represented as percentages (%) and number (n). SOFA: sequential organ failure assessment; APACHE: Acute Physiology and Chronic Health Evaluation. MV: mechanical ventilation; ICU: intensive care unit. ARDS: acute respiratory distress syndrome.*

**Table S3. Measurements at diagnosis based on 60-day in-hospital mortality.**

|  | **60-day Survivors** (n=81) | **60d nonsurvivors** (n=58) | **p-value** |
| --- | --- | --- | --- |
| **­Measurements at diagnosis,** [median [IQR]] |  |  |  |
| Mean arterial pressure (mmHg) | 66 [12] | 63.3 [11.3] | 0.300 |
| Hear rate (bpm) | 110 [30] | 120 [38] | **0.024** |
| PaCO_2_ (mmHg) | 39.9 [9.30] | 45 [11.6] | **0.007** |
| SaO_2_ (%) | 95.2 [7.1] | 95.1 [6.4] | 0.702 |
| PaO_2_ (mmHg) | 93 [43.3] | 84.1 [36.6] | 0.513 |
| pH | 7.35 [0.17] | 7.31 [0.15] | **<0.001** |
| HCO_3_ (mmol/L) | 21.8 [5.8] | 20.4 [7.2] | 0.126 |
| Creatinine mg/mL | 1.82 [1.91] | 2.27 [3.18] | **0.043** |
| LDH (UI) | 269.5 [260] | 354 [283] | 0.056 |
| Troponins (UI) | 77.58 [502] | 97.5 [519.7] | 0.434 |
| Lactate (mmol/L) | 2.2 [1.90] | 3 [2.9] | **0.010** |
| Procalcitonin (ng/ml) | 3.3 [13.7] | 9.9 [40.3] | **0.005** |
| C-Reactive Protein (mg/L) | 229 [190.5] | 255 [201] | 0.208 |
| White Blood cells (cells/mm^3^) | 15111 [9940] | 12245 [12880] | 0.388 |
| Compliance (mL/cmH_2_O) | 26.3 [22] | 22.7 [10] | 0.185 |
| Driving pressure (cmH_2_O) | 19 [11] | 20 [6] | 0.614 |

*Continuous variables are represented as median and interquartile range (IQR). LDH: Lactate Dehydrogenase*
